# Cerebrovascular regulation in men and women: stimulus-specific role of cyclooxygenase

**DOI:** 10.14814/phy2.12451

**Published:** 2015-07-06

**Authors:** Garrett L Peltonen, John W Harrell, Cameron L Rousseau, Brady S Ernst, Mariah L Marino, Meghan K Crain, William G Schrage

**Affiliations:** Bruno Balke Biodynamics Laboratory, Department of Kinesiology, University of Wisconsin-MadisonMadison, Wisconsin

**Keywords:** Brain blood flow, cerebral blood flow, hypercapnia, hypoxia, middle cerebral artery, sex

## Abstract

Greater cerebral artery vasodilation mediated by cyclooxygenase (COX) in female animals is unexplored in humans. We hypothesized that young, healthy women would exhibit greater basal cerebral blood flow (CBF) and greater vasodilation during hypoxia or hypercapnia compared to men, mediated by a larger contribution of COX. We measured middle cerebral artery velocity (MCAv, transcranial Doppler ultrasound) in 42 adults (24 women, 18 men; 24 ± 1 years) during two visits, in a double-blind, placebo-controlled design (COX inhibition, 100 mg oral indomethacin, Indo). Women were studied early in the follicular phase of the menstrual cycle (days 1–5). Two levels of isocapnic hypoxia (S_P_O_2_ = 90% and 80%) were induced for 5-min each. Separately, hypercapnia was induced by increasing end-tidal carbon dioxide (PET_CO__2_) 10 mmHg above baseline. A positive change in MCAv (ΔMCAv) reflected vasodilation. Basal MCAv was greater in women compared to men (*P* < 0.01) across all conditions. Indo decreased baseline MCAv (*P* < 0.01) similarly between sexes. Hypoxia increased MCAv (*P* < 0.01), but ΔMCAv was not different between sexes. Indo did not alter hypoxic vasodilation in either sex. Hypercapnia increased MCAv (*P* < 0.01), but ΔMCAv was not different between sexes. Indo elicited a large decrease in hypercapnic vasodilation (*P* < 0.01) that was similar between sexes. During the early follicular phase, women exhibit greater basal CBF than men, but similar vasodilatory responses to hypoxia and hypercapnia. Moreover, COX is not obligatory for hypoxic vasodilation, but plays a vital and similar role in the regulation of basal CBF (∼30%) and hypercapnic response (∼55%) between sexes.

## Introduction

Cerebrovascular disease is a leading cause of death in the United States and accounts for over $38 billion dollars in annual medical costs (Go et al. 2013). Stroke risk is highly sex-specific, as men exhibit a 33% greater incidence, 41% greater prevalence, and earlier onset of first-ever stroke than women (Appelros et al. 2009; Petrea et al. 2009). Cerebrovascular reactivity is also greater in women (Kastrup et al. 1997) and is associated with reduced stroke risk (Yonas et al. 1993). Despite these epidemiologic observations, there is a lack of mechanistic human studies investigating potential sex differences in cerebral blood flow (CBF) regulation.

Animal data indicate sex differences in CBF regulation. Isolated cerebral arteries from female rats exhibit larger diameters than males (Geary et al. 1998). The reduced cerebrovascular tone in female animal models is due, in part, to cyclooxygenase (COX) mediated production of the vasodilatory prostanoid, prostacyclin (Ospina et al. 2002, 2003; Krause et al. 2011). It should be noted, however, that animal studies utilize supraphysiologic sex hormone supplementation after gonadectomy, which does not accurately represent the internal environment in young, healthy humans.

Several human studies are consistent with observations seen in animals. Women display greater resting global CBF (Rodriguez et al. 1988; Esposito et al. 1996) and higher CBF velocities compared to men (Vriens et al. 1989; Martin et al. 1994; Olah et al. 2000; Krejza et al. 2005; Tegeler et al. 2013). Additionally, greater cerebrovascular reactivity in women has been suggested to be mediated by COX metabolites (Kastrup et al. 1999). Importantly, these studies did not control for menstrual phase or account for sex hormones that are known to acutely alter CBF and cerebrovascular responsiveness (Brackley et al. 1999; Krejza et al. 2001, 2003, 2004, 2013; Nevo et al. 2007). Without accounting for menstrual phase, it is difficult to differentiate whether greater CBF responsiveness in women is due to a fundamental sex difference, or acute fluctuations in sex hormones during the menstrual cycle.

Hypercapnia and hypoxia are environmental stressors that both elicit robust increases in CBF. Women demonstrate a greater hypercapnic increase in CBF than men (Karnik et al. 1996; Kastrup et al. 1997, 1999; Olah et al. 2000) that may be mediated by COX (Kastrup et al. 1999). However, as previously noted, designs that do not control for menstrual cycle or account for sex hormones limit interpretation. From a clinical perspective hypoxia may hold greater relevance than hypercapnia as it is characteristic of sleep disordered breathing, a condition noted for sex-specific pathophysiology (Jordan and McEvoy 2003). To our knowledge, no studies to date have examined sex differences in hypoxic vasodilation. Animal studies suggest COX contributes to hypoxic CBF regulation (Coyle et al. 1993; Fredricks et al. 1994), whereas in humans it appears that COX may (Hoiland et al. 2015) or may not play a substantial role in mediating hypoxic vasodilation (Fan et al. 2011; Harrell and Schrage 2014). Importantly, neither animal nor human studies have addressed potential sex differences in COX mediated regulation of hypoxic cerebral vasodilation. Human studies with physiologically relevant hormone levels and accounting for menstrual phase may provide direct translational insight into sex-specific regulation of CBF (Jickling and Sharp 2014).

Exploration of the sex-specific mechanisms responsible for the regulation of CBF is warranted to develop strategies that will slow or prevent the onset of cerebrovascular disease in both men and women. The primary purpose of this study was to investigate potential sex differences in CBF regulation during two distinct environmental stressors, hypoxia and hypercapnia. The secondary purpose of this study was to determine if the contribution of COX to the regulation of basal CBF, hypoxia, or hypercapnia differs between the sexes. We hypothesized women would exhibit greater basal CBF, greater vasodilation to hypoxia and hypercapnia, and a greater contribution of COX. Our rationale focused on comparing women during the early follicular phase of the menstrual cycle (when sex hormones least different from men) to understand fundamental sex difference in CBF regulation without the confounding effects of acute elevations in sex hormones. Results from this study will lay the framework for follow-up studies comparing across the menstrual cycle.

## Methods

### Subjects

A total of 42 young healthy adults were recruited to participate (18 men, 26 ± 1 years; 24 women, 23 ± 1 years). Subjects were free of disease, otherwise healthy, not currently taking medication with the exception of birth control, and sedentary (<120 min of moderate physical activity per week) as determined by health history and physical activity questionnaire. Women were not pregnant (urine pregnancy test) and studied during the early follicular phase of the menstrual cycle (days 1–5) or the low-hormone phase of birth control (birth control; *n* = 9). The experimental protocol conformed to the standards set forth by the Declaration of Helsinki and was approved by the University of Wisconsin-Madison Institutional Review Board. The nature, purpose, and risks of the study were provided to each subject before written informed consent was obtained. Data from a subset of subjects (men, *n* = 6; women, *n* = 6) were previously reported in a study comparing healthy controls to adults with metabolic syndrome (Harrell and Schrage 2014).

### Measurements

Height and weight were measured to calculate body mass index (BMI, kg m^−2^). Waist and hip circumferences were measured as indicators of regional adiposity. Venous blood samples were obtained for the determination of glucose, lipids, insulin, and sex hormones. During each visit, subjects were studied in a semirecumbent position and instrumented for continuous measurement of heart rate (3-lead ECG), pulse oximetry oxygen saturation (S_P_O_2_, pulse oximeter), and blood pressure (MABP, automated physiological monitor; GE Datex-Ohmeda, Madison, WI). A 2-MHz transcranial Doppler ultrasound probe (TCD, Neurovision model 500M, Multigon Industries, Inc.; Yonkers, NY) was placed over the right or left temporal window and after obtaining an optimal signal secured by an adjustable headband for measurement of middle cerebral artery velocity (MCAv) (Barnes et al. 2012; Harrell et al. 2013; Harrell and Schrage 2014; Smirl et al. 2014). Inspiratory and expiratory gases were measured with a gas analyzer (GEMINI, CWE, Inc., Ardmore, PA) and respiratory flow was determined with a heated pneumotachometer (Hans Rudolph Inc., Shawnee, KS).

### Protocol

**Figure 1:**
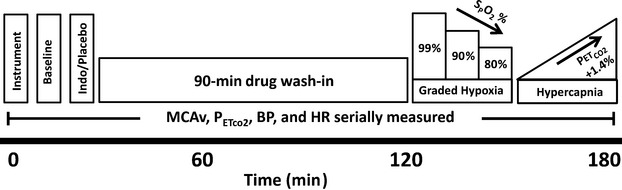
Timeline of study visits. Placebo and Indomethacin were administered in a randomized, double-blind order. Order of graded hypoxia and hypercapnia were randomized for each study visit. Indo, Indomethacin; S_P_O_2_, pulse oximetry oxygen saturation; PET_CO__2_, end-tidal carbon dioxide; MCAv, middle cerebral artery velocity; BP, blood pressure; HR, heart rate.

In a randomized, double-blind, and placebo-controlled design subjects completed two visits, under control (placebo) and experimental conditions (COX inhibition). Both placebo and the nonselective COX inhibitor Indomethacin (Indo, 100 mg, Heritage Pharmaceuticals Inc., Edison, NJ) were administered orally. Subjects reported to the laboratory after completing a 10-h fast and having abstained from exercise, alcohol, caffeine, and nonsteroidal anti-inflammatory drugs for a minimum of 18-h. After baseline data collection subjects received either placebo or Indo in addition to 20 mL of Maalox. Maalox was provided to prevent gastrointestinal discomfort occasionally associated with oral Indo. Subjects then rested quietly for 90-min while MCAv, respiratory, and cardiovascular variables were recorded for 5-min, spaced by 10-min nonrecording intervals. After 90-min, hypoxic and hypercapnic trials were conducted in randomized order while recording MCAv, respiratory, and cardiovascular variables during each trial. All trials were separated by 10-min of quiet rest while breathing room air (Fig.1). TCD placement was maintained during study visits and was similar between study visits.

### Hypoxia

Isocapnic hypoxia trials were performed as previously described in our laboratory (Limberg et al. 2012; Harrell et al. 2013; Harrell and Schrage 2014). Briefly, subjects inspired through a two-way nonrebreathing valve (2700 Series, Hans Rudolph Inc., Kansas City, MO), connected to a gas mixer (PM5300; Precision Medical, Northampton, PA), supplied by medical-grade pressurized oxygen (O_2_), carbon dioxide (CO_2_), and nitrogen (N_2_, Airgas, Madison, WI). After 5-min of baseline room air breathing, hypoxia was introduced by decreasing inspired O_2_ to elicit and sustain 5-min of S_P_O_2_ = 90%, followed immediately by a transition to and 5-min of S_P_O_2_ = 80%. Isocapnia was achieved through the addition of CO_2_ to inspired gas. PET_CO2_ has been shown to be a valid predictor of arterial blood CO_2_ levels (McSwain et al. 2010). Two women were unable to complete the S_P_O_2_ = 80% hypoxia trial due to hypoxia intolerance and were omitted from S_P_O_2_ = 80% hypoxia data analysis.

### Hypercapnia

Hypercapnic trials were performed as previously described in our laboratory (Harrell et al. 2013; Harrell and Schrage 2014). Briefly, subjects inspired through a three-way sliding rebreathing valve (Model 2870; Hans Rudolph Inc., Shawnee, KS) attached to a latex balloon containing a hyperoxic (O_2_ = 40%), hypercapnic (CO_2_ = 3%) gas mixture with the balance N_2_. The balloon was filled to a volume exceeding estimated vital capacity (as determined by age, sex, and height) by 1-L. After 5-min of baseline room air breathing, hypercapnia commenced and was sustained (∼2-min) until PET_CO2_ values reached 10 mmHg above baseline values. A total of two hypercapnic trials were completed and separated by 10-min of quiet rest and room air breathing. Values for the two trials were averaged.

### Plasma assays

Venous blood samples were drawn at baseline and after 90-min of placebo and Indo. Blood was centrifuged and plasma was drawn off and stored at −80°C. Circulating estrogen and insulin were determined with radioimmunoassay (RIA), while enzyme immunoassay (EIA) was utilized for the determination of progesterone, testosterone, and dihydrotestosterone.

### Data analysis

All cardiovascular and respiratory data were recorded, stored, and analyzed with PowerLab and LabChart (ADInsturments Inc., Dunedin, NZ). The effect of drug administration was examined over 90-min with the last 30-sec of each 5-min data recording interval used for analysis. During hypoxia, the last 30-sec of the 5-min recording intervals for baseline, S_P_O_2_ = 90%, and S_P_O_2_ = 80% were analyzed. Analysis of hypercapnia data included the last 30-sec of baseline and the last 10-sec of hypercapnia, equating to a 10 mmHg increase in PET_CO2_. Automated blood pressure recordings were taken during the last 30-sec of each recording interval to coincide with analysis intervals. The main outcome variable was MCAv, but to account for potential differences in perfusion pressure, cerebrovascular conductance index was also calculated and is presented in tables (CVCi = MCAv*100/MABP).

### Statistical analysis

Minitab 16 (State College, PA) was used for statistical analysis. Subject characteristics were compared using an unpaired student’s *t*-test. Significance of sex (men vs. women) and time (0, 15, 30, 45, 60, 75, and 90 min) on basal MCAv, with and without Indo, were determined utilizing a general linear model to perform two-way analysis of variance (ANOVA). Unpaired student’s *t*-tests were used to examine sex differences in the change in MCAv following Indo. Significance of sex (men vs. women) and hypoxia (baseline, S_P_O_2_ = 90%, and S_P_O_2_ = 80%) on MCAv and change in MCAv from baseline, with and without Indo, were determined by two-way general linear model ANOVA. Significance of sex (men vs. women) and hypercapnia (baseline and hypercapnia) on MCAv, with and without Indo, were determined by two-way general linear model ANOVA. Unpaired student’s t-tests were used to examine sex differences in the change in MCAv with hypercapnia following placebo and Indo. In a secondary analysis, the contribution of COX to hypoxic or hypercapnic mediated increases in MCAv was determined with general linear model ANOVA. Level of significance was set a priori at *P* < 0.05. When ANOVA yielded significance, multiple comparisons on factor means were performed with Tukey’s post hoc analysis. Data are expressed as mean ± standard error of the mean.

## Results

### Subjects

Subject characteristics are summarized in Table1. Men and women were similar in age and matched for activity level. Men had significantly greater height, weight, and BMI. Women had significantly greater total cholesterol and HDL. Values in both groups were considered healthy. Due to a lower body mass, women received a larger relative dose of Indo (*P* < 0.01). Circulating levels of sex hormones were determined in all 18 men and 20 of 24 women. Testosterone and DHT were greater in men (*P* < 0.01). By design, estrogen and progesterone were similar between sexes, confirming our menstrual phase selection criteria and allowing us to focus on sex differences when female sex hormones differences are minimized.

**Table 1:** Subject characteristics.

	Men	Women
	*n* = 18	*n* = 24
Age (year)	26 ± 1	23 ± 1
Height (cm)	178 ± 2	165 ± 1*
Weight (kg)	74 ± 2	58 ± 2*
BMI (kg m^−2^)	23 ± 1	21 ± 0*
Waist (cm)	83 ± 1	77 ± 3
Hip (cm)	101 ± 1	93 ± 2*
PAQ (kcal week^−1^)	1654 ± 294	1976 ± 311
Glucose (mg dL^−1^)	75 ± 2	75 ± 1
Insulin (*μ*U mL^−1^)	11 ± 1	10 ± 1
Total Cholesterol (mg dL^−1^)	141 ± 7	162 ± 5*
HDL (mg dL^−1^)	52 ± 3	71 ± 4*
LDL (mg dL^−1^)	75 ± 8	76 ± 4
Triglycerides (mg dL^−1^)	73 ± 8	73 ± 3
Systolic BP (mmHg)	118 ± 2	113 ± 2
Diastolic BP (mmHg)	74 ± 1	74 ± 1
MABP (mmHg)	89 ± 1	87 ± 1
Estradiol (pg mL^−1^)	58 ± 3	84 ± 25
Progesterone (pg mL^−1^)	409 ± 59	352 ± 46
Testosterone (pg mL^−1^)	4120 ± 251	315 ± 29*
DHT (pg mL^−1^)	552 ± 34	206 ± 15*
Indo Dose (mg kg^−1^)	1.4 ± 0	1.7 ± 0*

Values are presented as mean ± SE. Sex hormones (men, *n* = 18; women, *n* = 20). BMI, body mass index; PAQ, physical activity questionnaire; HDL, high-density lipoprotein; LDL, low-density lipoprotein; BP, blood pressure; MABP, mean arterial blood pressure; DHT, dihydrotestosterone; Indo, indomethacin.

* Men versus women; *P* < 0.05.

### Basal cerebral blood flow: sex comparison

**Figure 2:**
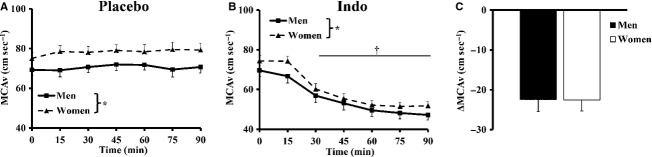
Basal middle cerebral artery velocity (MCAv) following placebo or indomethacin (Indo) administration. (A) MCAv was greater in women over the 90-min of placebo wash-in. (B) MCAv was decreased by 30-min of Indo wash-in, but remained greater in women. (C) The absolute change in MCAv (ΔMCAv) 90-min following Indo was similar in both sexes. *Main effect of sex; †main effect of Indo; *P* < 0.01.

Cardiorespiratory variables collected prior to and serially for 90-min after the administration of placebo while breathing normal room air are presented in Table2. Men had greater MABP and PET_CO2_ than women (*P* < 0.05). Basal MCAv was greater in women (Fig.2A; *P* < 0.01). During placebo, MCAv did not change over the course of 90-min.

**Table 2:** Cerebrovascular and cardiorespiratory variables prior to and for 90-min following Placebo or Indo administration.

	Placebo		Indo	
	Men	Women	Men	Women
MABP (mmHg)^1^,^2^				
Baseline	88 ± 1	86 ± 1	90 ± 2	86 ± 2
30 min	90 ± 2	87 ± 1	94 ± 2	90 ± 1
60 min	89 ± 2	87 ± 2	92 ± 2	93 ± 1
90 min	89 ± 1	86 ± 1	94 ± 2	91 ± 1
PET_co2_ (mmHg)^1^,^2^				
Baseline	38 ± 1	36 ± 1	38 ± 1	37 ± 1
30 min	39 ± 0	37 ± 1	38 ± 0	37 ± 0
60 min	39 ± 0	38 ± 1	38 ± 0	37 ± 1
90 min	38 ± 1	38 ± 1	38 ± 1	37 ± 1
SpO_2_ (%)				
Baseline	99 ± 0	99 ± 0	99 ± 0	99 ± 0
30 min	99 ± 0	99 ± 0	99 ± 0	99 ± 0
60 min	99 ± 0	99 ± 0	99 ± 0	99 ± 0
90 min	99 ± 0	99 ± 0	99 ± 0	99 ± 0
MCAv (cm sec^−1^)^1^,^2^,^3^				
Baseline	69 ± 3	75 ± 3	70 ± 3	74 ± 3
30 min	71 ± 3	78 ± 3	57 ± 3	60 ± 3
60 min	72 ± 3	78 ± 4	50 ± 3	52 ± 2
90 min	71 ± 3	79 ± 3	47 ± 3	52 ± 2
ΔMCAv				
90 min	1 ± 1	4 ± 2	22 ± 2	23 ± 2
% ΔMCAv				
90 min	2 ± 2	6 ± 3	32 ± 3	30 ± 2
CVCi (cm sec^−1^ mmHg^−1^)^1^,^2^,^3^				
Baseline	79 ± 3	88 ± 4	78 ± 4	87 ± 3
30 min	79 ± 3	90 ± 4	61 ± 4	67 ± 3
60 min	81 ± 3	91 ± 4	54 ± 3	57 ± 3
90 min	80 ± 3	92 ± 4	50 ± 3	57 ± 2
Δ CVCi				
90 min	1 ± 2	4 ± 3	28 ± 3	30 ± 3
% Δ CVCi				
90 min	1 ± 2	6 ± 4	35 ± 3	34 ± 3

Values are presented as mean ± SE. Indo, indomethacin; MABP, mean arterial blood pressure; PET_CO2_, end-tidal carbon dioxide; S_P_O_2_, pulse oximetry oxygen saturation; MCAv, middle cerebral artery velocity; ΔMCAv, absolute change in MCAv; %ΔMCAv, relative change in MCAv; CVCi, cerebrovascular conductance index; ΔCVCi, absolute change in CVCi; %ΔCVCi, relative change in CVCi.

1 Placebo main effect of group.

2 Indo main effect of group.

3 Indo main effect of time; *P* < 0.05.

### Basal cerebral blood flow: contribution of COX

Cardiorespiratory variables collected prior to and serially for 90-min after the administration of Indo, while breathing normal room air are presented in Table2. Men had greater MABP and PET_CO2_ than women (*P* < 0.01). Indo increased MABP over the course of Indo wash-in (*P* < 0.01). MCAv decreased by the 30-min time point (*P* < 0.01) and remained suppressed for the reminder of the 90-min following Indo (Fig.2B). Women displayed higher MCAv than men during 90-min of Indo wash-in (Fig.2B; *P* < 0.01). The absolute change in MCA (ΔMCAv) with Indo was not different between sexes (Fig.2C).

### Hypoxia-mediated cerebral vasodilation: sex comparison

**Figure 3:**
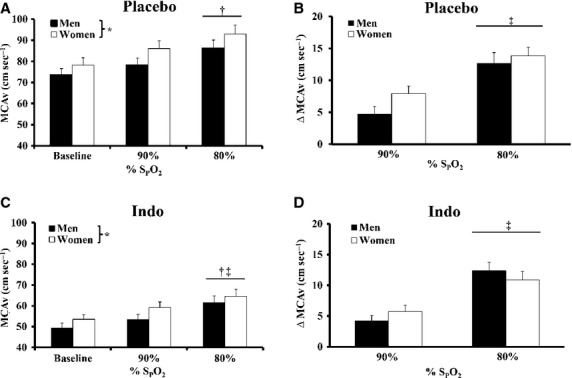
Middle cerebral artery velocity (MCAv) during hypoxia with placebo and indomethacin (Indo). (A) Hypoxia increased MCAv at S_P_O_2_ 80% and MCAv was greater in women with placebo. (B) The change in MCAv (ΔMCAv) was greater at S_P_O_2_ 80% than 90% with placebo, but ΔMCAv was not different between groups. (C) Hypoxia increased MCAv at S_P_O_2_ 80% with Indo and MCAv was greater in women. (D) ΔMCAv was not different between sexes during hypoxia with Indo, but ΔMCAv was greater at S_P_O_2_ 80% than 90%. *Main effect of sex, *P* < 0.05; †80% versus baseline, *P* < 0.05; ‡80% versus 90%, *P* < 0.01.

Hemodynamic and gas exchange variables collected during hypoxia are presented in Table3. Hypoxia reduced S_P_O_2_ (*P* < 0.01), but was not different between sexes. Hypoxia increased heart rate and MABP (*P* < 0.01). PET_CO2_ did not change during hypoxia, but was greater in men (*P* < 0.01). MCAv was not significantly different from baseline at S_P_O_2_ = 90%, but was increased at S_P_O_2_ = 80% (Fig.3A; *P* < 0.01). Accordingly, the ΔMCAv from baseline was greater at SpO_2_ = 80% than SpO_2_ = 90%, (Fig.3B; *P* < 0.01). MCAv was greater in women during hypoxia, but ΔMCAv was not different between sexes (Fig.3B).

**Table 3:** Cerebrovascular and cardiorespiratory variables during graded systemic hypoxia.

	Placebo		Indo	
	Men	Women	Men	Women
MABP (mmHg)^2^,^3^				
Baseline	90 ± 1	89 ± 1	95 ± 2	94 ± 1
90%	94 ± 1	93 ± 2	97 ± 2	94 ± 1
80%	94 ± 1	91 ± 2	98 ± 2	95 ± 1
HR (beats min^−1^)^1^,^2^,^4^				
Baseline	65 ± 5	70 ± 2	67 ± 6	62 ± 2
90%	75 ± 4	82 ± 2	70 ± 4	71 ± 2
80%	79 ± 3	89 ± 2	73 ± 2	76 ± 3
PET_co2_ (mmHg)^1^				
Baseline	40 ± 1	38 ± 0	38 ± 1	37 ± 1
90%	40 ± 1	38 ± 0	38 ± 1	38 ± 0
80%	39 ± 1	38 ± 0	38 ± 1	38 ± 1
SpO_2_ (%)^2^,^4^				
Baseline	99 ± 0	99 ± 0	99 ± 0	99 ± 0
90%	90 ± 0	90 ± 0	90 ± 0	90 ± 0
80%	80 ± 0	81 ± 0	80 ± 0	81 ± 0
MCAv (cm sec^−1^)^1^,^2^,^3^,^4^				
Baseline	73 ± 3	78 ± 3	49 ± 2	54 ± 2
90%	78 ± 3	86 ± 4	53 ± 3	59 ± 3
80%	86 ± 4	93 ± 4	62 ± 3	65 ± 3
ΔMCAv^2^,^4^				
90%	5 ± 1	8 ± 1	4 ± 1	6 ± 1
80%	13 ± 2	14 ± 1	12 ± 1	11 ± 1
% ΔMCAv 2,4				
90%	7 ± 2	10 ± 2	9 ± 2	11 ± 2
80%	17 ± 2	18 ± 2	25 ± 3	20 ± 2
CVCi (cm sec^−1^ mmHg^−1^)^1^,^2^,^3^,^4^				
Baseline	82 ± 4	88 ± 4	52 ± 3	57 ± 2
90%	84 ± 4	93 ± 4	55 ± 3	63 ± 3
80%	92 ± 4	102 ± 4	63 ± 3	68 ± 4
Δ CVCi^2^,^4^				
90%	2 ± 2	5 ± 1	3 ± 1	6 ± 1
80%	10 ± 2	13 ± 1	11 ± 2	11 ± 2
% Δ CVCi^2^,^4^				
90%	2 ± 2	6 ± 2	7 ± 2	11 ± 2
80%	13 ± 2	16 ± 2	22 ± 3	18 ± 2

Values are means ± SE. Indo, indomethacin; MABP, mean arterial blood pressure; HR, heart rate; PET_CO2_, end-tidal carbon dioxide; S_P_O_2_, pulse oximetry oxygen saturation; MCAv, middle cerebral artery velocity; ΔMCAV, change in MCAv from baseline; %ΔMCAV, percent change in MCAv from baseline; CVCi, cerebrovascular conductance index; ΔCVCi, change in CVCi from baseline; %ΔCVCi, percent change in CVCi from baseline.

1 Placebo, main effect of group.

2 Placebo, main effect of hypoxia.

3 Indo, main effect of group.

4 Indo, main effect of hypoxia; *P* < 0.05.

### Hypoxia-mediated cerebral vasodilation: contribution of COX

Hemodynamic and gas exchange variables collected during hypoxia with Indo are presented in Table3. Hypoxia reduced S_P_O_2_ (*P* < 0.01), but SpO_2_ was not different between sexes. MCAv increased significantly at S_P_O_2_ = 80%, but not S_P_O_2_ = 90% (Fig.3C; *P* < 0.01). Accordingly, ΔMCAv from baseline was greater at SpO_2_ = 80% than SpO_2_ = 90% (Fig.3D; *P* < 0.01). MCAv was greater (Fig.3C; main effect, *P* < 0.05) and MABP was lower in women during hypoxia with Indo. The ΔMCAv during hypoxia with Indo was not different between sexes (Fig.3D) and was not different from the placebo condition. PET_CO2_ did not change during hypoxia (Table3) and was not different between sexes.

### Hypercapnia-mediated cerebral vasodilation: sex comparison

**Figure 4:**
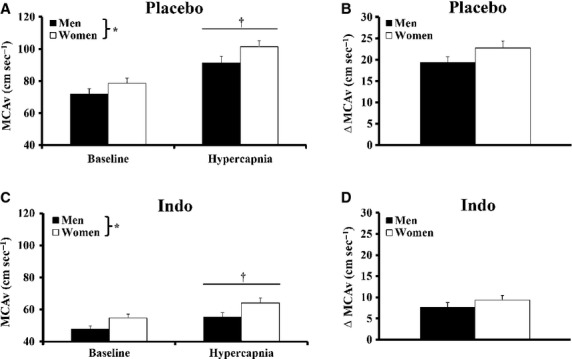
Middle cerebral artery velocity (MCAv) during hypercapnia with placebo and indomethacin (Indo). (A) Hypercapnia increased MCAv and MCAv was greater in women with placebo. (B) The change in MCAv (ΔMCAv) with hypercapnia was not different between groups. (C) Hypercapnia increased MCAv with Indo and MCAv was greater in women. (D) ΔMCAv was not different between sexes during hypercapnia with Indo. *Main effect of sex, *P* < 0.05; †hypercapnia versus baseline, *P* < 0.01.

Hemodynamic and gas exchange variables collected during hypercapnia are presented in Table4. By design, hypercapnia increased PET_CO2_ (*P* < 0.01). MCAv increased during hypercapnia (Fig.4A; *P* < 0.01) and was greater in women (Fig.4A; *P* < 0.05). However, ΔMCAv from baseline was not different between sexes (Fig.4B). PET_CO2_ was greater in men, but the change in PET_CO2_ was not different between sexes.

**Table 4:** Cerebrovascular and cardiorespiratory variables during hypercapnia.

	Placebo		Indo	
	Men	Women	Men	Women
MABP (mmHG)				
Baseline	90 ± 1	87 ± 1	95 ± 2	91 ± 2
Hypercapnia	92 ± 1	89 ± 2	96 ± 2	94 ± 2
HR (beats min^−1^)				
Baseline	65 ± 6	69 ± 2	62 ± 6	63 ± 2
Hypercapnia	65 ± 5	64 ± 3	61 ± 6	62 ± 2
PET_CO2_ (mmHG)^1^,^2^,^4^				
Baseline	39 ± 1	38 ± 0	38 ± 1	37 ± 0
Hypercapnia	49 ± 1	47 ± 0	48 ± 1	47 ± 0
S_P_O_2_ (%)^2^,^4^				
Baseline	99 ± 0	99 ± 0	99 ± 0	99 ± 0
Hypercapnia	100 ± 0	100 ± 0	100 ± 0	100 ± 0
MCAv (cm sec^−1^)^1^,^2^,^3^,^4^				
Baseline	72 ± 3	79 ± 3	48 ± 2	55 ± 2
Hypercapnia	91 ± 4	101 ± 4	55 ± 3	64 ± 3
ΔMCAv				
Hypercapnia	19 ± 1	23 ± 2	8 ± 1	9 ± 1
%ΔMCAv				
Hypercapnia	27 ± 2	30 ± 2	16 ± 2	17 ± 2
CVCi (cm sec^−1^ mmHG^−1^)^1^,^2^,^3^,^4^				
Baseline	80 ± 3	90 ± 4	50 ± 2	60 ± 2
Hypercapnia	100 ± 4	114 ± 4	58 ± 3	69 ± 3
Δ CVCi				
Hypercapnia	20 ± 2	23 ± 2	8 ± 1	9 ± 1
%Δ CVCi				
Hypercapnia	25 ± 2	27 ± 2	15 ± 2	14 ± 2

Values are means ± SE. Indo, indomethacin; MABP, mean arterial blood pressure; HR, heart rate; PET_CO2_, end-tidal carbon dioxide; S_P_O_2_, pulse oximetry oxygen saturation; MCAv, middle cerebral artery velocity; ΔMCAV, change in MCAv from baseline; %ΔMCAV, percent change in MCAv from baseline; CVCi, cerebrovascular conductance index; ΔCVCi, change in CVCi from baseline; %ΔCVCi, percent change in CVCi from baseline.

1 Placebo, main effect of group.

2 Placebo, main effect of hypercapnia.

3 Indo, main effect of group.

4 Indo, main effect of hypercapnia; *P* < 0.05.

### Hypercapnia-mediated cerebral vasodilation: contribution of COX

Hemodynamic and gas exchange variables collected during hypercapnia with Indo are presented in Table4. By design, hypercapnia increased PET_CO2_ (*P* < 0.01). MCAv increased during hypercapnia (Fig.4C; *P* < 0.01) and was greater in women (Fig.4C; *P* < 0.01). The ΔMCAv with Indo was lower than placebo hypercapnia (∼55% reduction, *P* < 0.01), but ΔMCAv was not different between sexes (Fig.4D). Change in PET_CO2_ was not different between sexes.

### Alternative data expression

CBF data are expressed as MCAv or ΔMCAv. Normalizing MCAv for perfusion pressure (MABP) and expressed as CVCi yielded the same results. Additionally, data expressed as relative changes in MCAv from baseline did not alter the conclusions. One exception was the %ΔMCAv and %ΔCVCi during hypoxia with Indo. Despite similar ΔMCAv during hypoxia with placebo or Indo, decreased baseline MCAv with Indo resulted in significantly greater relative ΔMCAv compared to placebo. As Indo substantially reduced basal MCAv, but did not change hypoxic absolute ΔMCAv, expressing data as relative change from baseline is inappropriate and does not provide additional insight into our findings.

There were 24 women who participated in this study, of which nine were on birth control and 15 were not. Comparing the hypoxic and hypercapnic responses between women on birth control and not on birth control revealed no differences in Placebo or Indo trials. When women on birth control were excluded from analysis between sexes (men, *n* = 18; women, *n* = 15), similar conclusions were reached, and so they were included in analysis (men, *n* = 18; women, *n* = 24).

## Discussion

The purpose of this study was to determine the fundamental sex difference in CBF regulation between men and women without the confounding factor of acute elevations in sex hormones associated with the female menstrual cycle. Our aim was to determine the sex-specific contribution of COX to cerebrovascular control. The findings of our investigations indicate: (1) there is a similar and substantial contribution (30%) of COX to basal CBF in both sexes; (2) the increase in CBF to hypoxia or hypercapnia is not different between sexes; (3) the contribution of COX to the hypoxic increase in CBF is minimal and similar between sexes; and (4) the contribution of COX to hypercapnic vasodilation is substantial (55%) and similar between sexes. Taken together, data suggest women exhibit greater basal CBF, but similar hypoxic and hypercapnic mediated vasodilation when sex hormone profiles are most comparable to men.

### Sex differences in basal CBF regulation

Previous TCD studies indicate premenopausal women exhibit higher basal MCAv than age-matched men (Vriens et al. 1989; Brouwers et al. 1990; Martin et al. 1994; Marinoni et al. 1998; Krejza et al. 2005; Tegeler et al. 2013) and our data support this concept. The average basal MCAv measured for men and women in our study over the course of 90-min was found to be approximately 70 cm sec^−1^ and 80 cm sec^−1^, respectively. These are nearly identical to previously published TCD values (Vriens et al. 1989; Krejza et al. 2005), lending confidence in our ability to accurately measure MCAv with TCD and compare sexes. In the context of comparing our present findings to studies lacking control of menstrual phase, we provide clear evidence that when estrogen and progesterone (Table1) are comparable to that of men, women have fundamentally higher basal CBF.

Estrogen increases cerebral artery vasodilation partially through stimulating production of the COX metabolite prostacyclin (Ospina et al. 2002, 2003; Sobrino et al. 2010). With this in mind, we hypothesized COX would have a greater contribution to basal CBF in women, potentially related to a greater chronic estrogen exposure than men. Contrary to our hypothesis, COX contributes significantly (∼30%) and equally to the maintenance of basal CBF in men and women (Fig.2). This reduction in resting CBF with COX inhibition is in accord with the previous work (Kastrup et al. 1999; Fan et al. 2011; Barnes et al. 2012; Harrell and Schrage 2014) and is supported when comparing absolute and relative reductions in basal MCAv. Interestingly after COX inhibition, there still remains an unidentified vasodilator mechanism maintaining a slightly greater resting CBF in women (Fig.2B).

### Hypoxia

Hypoxia is clinically relevant. Sleep disordered breathing is characterized by hypoxia and is noted for sex-specific pathophysiology (Jordan and McEvoy 2003). This is the first study to demonstrate hypoxic vasodilation is not different between men and women (Fig.3). Furthermore, we provide clear evidence COX does not contribute to hypoxic vasodilation in either sex (Fig.3). This supports previous studies (not examining sex differences) indicating COX does not play an obligatory role in regulating hypoxic vasodilation in humans, at least when lowering S_P_O_2_ to 80% (Fan et al. 2011; Harrell and Schrage 2014).

### Hypercapnia

Our new data indicate women exhibit similar CO_2_ reactivity compared to men, which contrasts with prior studies suggesting greater CO_2_ reactivity in women (Karnik et al. 1996; Kastrup et al. 1997, 1999; Olah et al. 2000). The higher CO_2_ reactivity reported in prior studies might be explained by the lack of control for menstrual phase and fluctuations in female sex hormones, which were not reported previously (Karnik et al. 1996; Kastrup et al. 1997, 1999; Olah et al. 2000). Along these lines, cerebrovascular reactivity to breath holding (CO_2_ accumulation) is greater in women during the luteal phase compared to the follicular phase of the menstrual cycle (Diomedi et al. 2001). Furthermore, sex hormone oscillations in younger women and hormone replacement therapy in older women are known to alter cerebrovascular reactivity to hypercapnia (Belfort et al. 1995; Kastrup et al. 1998; Krejza et al. 2013). In the current study, women were studied during the early follicular phase of the menstrual cycle when circulating sex hormones are least different from men (Table1). This likely explains the absence of a sex difference in CO_2_ reactivity (Fig.4). Taken in context with previous studies, fundamental sex differences in CO_2_ reactivity do not exist between healthy young men and women in the early follicular phase. Rather, greater CO_2_ reactivity in women during late follicular or luteal phases of menstrual cycle is likely explained by cyclic increases in circulating female sex hormones.

Cyclooxygenase inhibition in this study reduced hypercapnic vasodilation by ∼55% which is similar to that seen in prior studies not focused on sex comparisons (Barnes et al. 2012; Harrell and Schrage 2014). Although there was a robust decrease in hypercapnic vasodilation with COX inhibition, the contribution of COX was not different between sexes (Fig.4). Contrary to our findings, Kastrup et al. demonstrated women had greater CO_2_ reactivity that was abolished with COX inhibition when menstrual phase was not controlled (Kastrup et al. 1999). By controlling for menstrual cycle phase and quantifying circulating sex hormone levels, our new findings strongly suggest men and women demonstrate similar vasodilation to hypercapnia, as well as a similar mechanistic contribution of COX eliciting this response.

### Limitations

This study included a large number of well characterized subjects, controlled for menstrual cycle, quantified sex hormone levels, tightly controlled experimental conditions, and was a double-blind, randomized research design with multiple physiologic stressors. Given the strengths, there are limitations that need consideration. First, measuring MCAv with TCD is an estimation of CBF, as it is assumed that middle cerebral artery (MCA) diameter does not change. Based upon the levels of hypoxia and hypercapnia used in this study, MCA diameter likely remains constant (Poulin and Robbins 1996; Serrador et al. 2000; Wilson et al. 2011). MCA diameter has been shown to change during hypoxic challenges more extreme than those used in this study (Wilson et al. 2011) and during hypercapnia (Coverdale et al. 2014). There is no evidence to indicate that these diameter changes are sex-specific, therefore if MCA diameter were to change in response to our stressors we would be underestimating CBF (Coverdale et al. 2014) equally in both sexes. However, sex-specific MCA dilation cannot be completely ruled out and could contribute to our lack of sex difference. Secondly, we only measured CBF through one MCA but previous studies demonstrate interhemispheric asymmetry is lowest in MCA resulting in insignificant differences in MCAv between left and right sides (Vriens et al. 1989; Martin et al. 1994). Third, although we did not measure plasma metabolites of COX to test efficacy of COX inhibition, we previously reported a large and similar decrease in circulating COX metabolites, between two groups receiving different relative doses, coupled with large decreases in basal CBF and hypercapnic responses (Harrell and Schrage 2014). Furthermore, the robust decrease in basal CBF 30-min after Indo administration and the ∼55% decrease in hypercapnic responses in both sexes indicate COX was inhibited. Fourth, women in our group received a larger relative dose of Indo due to a smaller body size. Despite the greater relative dose, there was an ∼30% decrease in basal CBF in both sexes which is similar to that seen during Indo administration relative to body weight (Hoiland et al. 2015). A larger relative dose of Indo in women suggests we did not underestimate the hypothesized greater vasodilation in women and further supports our conclusions. Fifth, our findings are limited by the comparison of men to women during the early follicular phase of the menstrual cycle, which we sought to explore the basic sex difference in CBF regulation when sex hormones are most similar. An intriguing follow-up investigation should focus on whether acute elevations in female sex hormones in varying phases of the menstrual cycle and these hormonal influence CBF regulation and the contribution of COX.

## Summary and Conclusion

We systematically tested the hypothesis that women would exhibit greater basal CBF, greater cerebral vasodilation to hypercapnia and hypoxia, and greater contribution of COX. Our findings establish women in the early follicular phase of the menstrual cycle exhibit greater basal CBF compared to men. New findings demonstrate hypoxia and hypercapnia mediated increases in CBF are remarkably similar between sexes. Additionally, COX does not appear to contribute to hypoxic vasodilation in either men or women. Finally, our results indicate COX contributes substantially to both basal CBF and hypercapnic vasodilation, and this contribution is similar between sexes. Taken together, these data establish fundamental insight into CBF regulation that can be used to design mechanistic studies to unravel the complex sex-age-hormone interactions seen in overt clinical cerebrovascular disease developing during middle to old age.
